# Association Between Resistance to Cinacalcet and Parathyroid Gland Hyperplasia in Kidney Transplant Recipients with Persistent Hypercalcemia

**Published:** 2020

**Authors:** A. Oruc, A. Ersoy, A. A. Kocaeli, A. Yildiz, O. O. Gul, E. Ertürk, C. Ersoy

**Affiliations:** 1 *Uludag University Faculty of Medicine, Department of Nephrology, Bursa, Turkey*; 2 *Uludag University Faculty of Medicine, Department of Endocrinology, Bursa, Turkey*

**Keywords:** Kidney transplantation, Hypercalcemia, Cinacalcet, Parathyroid adenoma, Parathyroidectomy

## Abstract

**Background:**

Persistent hypercalcemia and hyperparathyroidism after successful kidney transplantation can be detrimental in some recipients and should be ameliorated.

**Objective:**

To point out the concerns regarding resistance to cinacalcet in kidney transplant recipients with persistent hypercalcemia.

**Methods:**

14 renal transplant recipients who received cinacalcet treatment because of persistent hypercalcemia were included in the study. Serum creatinine, estimated glomerular filtration rate (eGFR), calcium, phosphorus, and intact parathyroid hormone (PTH) levels at the baseline and throughout the treatment, and ultrasonography and parathyroid scintigraphy findings were recorded.

**Results:**

Cinacalcet treatment was initiated after a mean±SD of 20.7±19.7 months of transplantation and maintained for 16.9±7.9 months. Serum calcium levels were significantly decreased with the cinacalcet treatment. There were no significant changes in serum creatinine, eGFR, phosphorus, and PTH levels. In all participants, serum calcium levels were increased from 9.8±0.6 to 11.1±0.6 mg/dL (p<0.001) within 1 month of cessation of cinacalcet. 7 recipients with adenoma-like hyperplastic glands underwent parathyroidectomy (PTx) due to failure with cinacalcet.

**Conclusion:**

Cinacalcet may be an appropriate treatment for a group of recipients with hypercalcemia without adenoma-like hyperplastic glands or who had a contraindication for surgery. Recipients with enlarged parathyroid gland may resist to cinacalcet-induced decrease in serum PTH, although the concomitant hypercalcemia may be corrected.

## INTRODUCTION

Patients with end-stage renal disease experience clinical disorders related to mineral and bone disease after kidney transplantation; those include calcium (Ca) and phosphorus (P) disorders, vitamin D deficiency, osteoporosis, and secondary and tertiary hyperparathyroidism [1]. Although serum Ca, P and intact parathyroid hormone (PTH) levels tend to normalize over the time after transplantation [2, 3], hypercalcemia has been reported in 5%–66% of recipients [4-6]. Besides, one-third of recipients have elevated PTH levels at six months of transplantation and even after the first year [3]. Persistent hyperparathyroidism (PHPT) is the leading cause of persistent hypercalcemia. In our previous study, the prevalence of hypercalcemia and PHPT at 12 months post-transplantation were 19% and 53.3%, respectively [7]. The prevalence of hypercalcemia in patients who had moderate to severe hyperparathyroidism prior to kidney transplantation can be higher [1]. Persistent hypercalcemia leads to several detrimental effects on graft and recipient as nephrolithiasis and nephrocalcinosis, poor graft function, and adverse cardiovascular events [8-10]. Effective treatment of persistent hypercalcemia is considered to provide better graft and recipient outcome. 

Cinacalcet is a calcimimetic drug which is approved for primary and secondary hyperparathyroidism. It is also an effective and safe treatment for hypercalcemia due to PHPT in kidney transplant recipients with off-label permission [11-14]. Although serum Ca and PTH levels decrease during cinacalcet treatment, there are concerns about treatment duration due to re-elevation of serum Ca levels and PTH levels to pre-treatment period after cessation [15-18]. We conducted this study to evaluate the efficacy of cinacalcet treatment and point out the concerns regarding resistance to cinacalcet in kidney transplant recipients with persistent hypercalcemia.

## MATERIALS AND METHODS

This retrospective study analyzed the data of 390 consecutive adult patients who underwent kidney transplantation between January 2008 and January 2015. PHPT is defined as serum corrected Ca level of >10.2 mg/dL (at least twice in a 6-month period) and PTH >150 pg/mL 6^th^ month post-transplantation. We identified 15 recipients who received cinacalcet treatment with off-label permission for hypercalcemia due to PHPT. They agreed to enter the study and signed an informed written consent. One of the 15 patients was excluded from the study due to missing data. 

We retrieved demographic and laboratory (serum creatinine, eGFR, Ca, P, and PTH levels) before transplantation and at the baseline and 1^st^, 3^rd^, 6^th^, and 12^th^ months and the last visit of the cinacalcet treatment from the database in our electronic files. eGFR was calculated by the Chronic Kidney Disease Epidemiology Collaboration (CKD-EPI) formula [19]. Ultrasonography (USG) and parathyroid scintigraphy were performed for all recipients at the baseline of cinacalcet treatment. Recipients received 30 mg/day cinacalcet initially. Cinacalcet dose was then modified according to laboratory findings at the visits and increased in five recipients to achieve a serum Ca level <10.2 mg/dL. The efficacy of the treatment was evaluated at 6^th^ and 12^th^ months of cinacalcet therapy. Recipients resistant to cinacalcet or who could not get off-label permission for cinacalcet were re-evaluated for parathyroidectomy (PTx). 

Ethics

Ethical approval was obtained by the Ethics Committee of the Uludag University, Faculty of Medicine.

Statistical Analysis

SPSS^®^ for Windows^®^ ver 13.0 (SPSS, Inc, Chicago, IL, USA) was used for data analyses. For continuous variables, the results were expressed as mean±SD. The Wilcoxon signed rank test was used for comparisons within the group. A p value <0.05 was considered statistically significant. 

## RESULTS

The mean±SD age of 14 studied patients (12 deceased, 2 living donor; 7 males and 7 females) was 47.1±9.8 years. The mean±SD dialysis duration was 110±43 months; the follow-up period after transplantation was 79.5±27.2 months. The cinacalcet treatment was started on a mean±SD of 20.7±19.7 months post- transplantation. The mean±SD duration of cinacalcet treatment was 16.9±7.9 months. Compared to the baseline levels, there were significant (p<0.001) decreased in serum Ca levels measured at the 1^st^, 3^rd^, 6^th^, and 12^th^ months of the cinacalcet treatment and at last visit. There were no significant changes in serum creatinine, eGFR, and PTH levels throughout the study period. Serum P levels increased significantly with cinacalcet treatment compared with the baseline and values measured at the 1^st^ (p=0.043), 3^rd^ (p=0.011), and 6^th^ (p=0.004) months of the cinacalcet treatment, and also the last visit (p=0.029) (Table 1). Parathyroid scintigraphy revealed parathyroid adenoma in eight recipients, parathyroid hyperplasia in one and normal findings in five. Parathyroid adenoma was identified with USG in eight recipients who also had adenoma in scintigraphy. USG revealed normal findings in six recipients. 

**Table 1:** The changes in laboratory findings of 14 recipients during cinacalcet therapy

Lab Parameter	Baseline	Month 1	Month 3	Month 6	Month 12	1 month after cessation	Last visit
Serum calcium (mg/dL)	11.3±0.4	10.3±0.8^a^	9.8±0.8^a^	9.9±0.5^a^	9.8±0.6^a^	11.3±0.8^b^	10±0.7^c^
Serum phosphorus (mg/dL)	2.5±0.7	2.9±0.5^d^	2.9±0.5^d^	3.1±0.6^d^	2.5±0.5	2.5±0.5	2.9±0.5^d^
Serum PTH (pg/mL)	386±237	337±180	356±193	442±362	494±395	540±730	307±195
Serum creatinine (mg/dL)	1.2±0.4	1.2±0.3	1.2±0.4	1.1±0.3	1.2±0.4	1.3±0.4	1.2±0.4
eGFR (mL/min/1.73 m^2^)	71.1±28.4	72.1±25.9	72±25	74.4±23.7	73.2±26.1	61.5±29.5	68.5±24.4

PTH: Intact parathyroid hormone; eGFR: Estimated glomerular filtration rate

^a^p<0.001 compared with the baseline; ^b^p<0.001 compared with month 12; ^c^p=0.001 compared with the baseline; ^d^p<0.05 compared with the baseline

Cinacalcet was approved with off-label permission, and there was a compulsory break in treatment at the 12^th^ month because of taking time of documents approval. In cinacalcet-free period, Ca levels increased from 9.8±0.6 to 11.1±0.6 mg/dL (p<0.001) within one month in all recipients. 

Although serum Ca levels decreased with cinacalcet, we decided that PTx could provide permanent amelioration for eight recipients with adenoma-like hyperplastic glands in whom Ca levels increased after withdrawal of cinacalcet. Seven of eight recipients underwent PTx; the remaining one had to continue to use cinacalcet because of contraindication for surgery due to bilateral carotid artery occlusion. In two recipients, a second PTx was performed because of residual parathyroid tissue. Normal serum Ca levels were provided for all recipients after PTx. Histopathologic evaluation revealed nodular hyperplasia in five of seven and adenoma in other two recipients. After PTx, two recipient returned to hemodialysis because of chronic allograft nephropathy who had had stage 4 chronic kidney disease before PTx. 

Six of 14 recipients continued to use cinacalcet; their parathyroid scintigraphy revealed parathyroid hyperplasia in one and normal findings in five. However, PTx was chosen in three of six who had high serum Ca levels (10.7, 11.1, and 11.2 mg/dL) and PTH levels (550, 354, and 352 pg/mL) despite cinacalcet treatment. At their last visit, only two of six had normal serum Ca levels of 10.1 and 9.8 mg/dL under cinacalcet treatment for 19 and 36 months. Only serum Ca levels of one patient were in the normal range near to the upper normal limits (10 mg/dL) without cinacalcet treatment; the patient had normal parathyroid scintigraphy and USG findings. 

## DISCUSSION

Persistent hypercalcemia and PHPT should be treated because of graft dysfunction [5, 8, 20]. The treatment options in these patients are PTx or temporarily treatment with cinacalcet with dose titration [21]. If the patient does not have hypercalcemia-associated graft dysfunction, many transplant centers prefer to wait for at least a year after kidney transplantation before proceeding to surgery, because of spontaneous involution of hyperplastic parathyroid glands can occur over a year that leads to a decline in PTH level. In our study, cinacalcet treatment reduced serum Ca levels safely and effectively, similar to the results of other studies [11, 12, 21-24]. Cinacalcet increases the sensitivity of Ca sensing receptors of the parathyroid gland and decreases PTH and Ca levels [25]. Cinacalcet is effective throughout the time it is used. However, cinacalcet treatment is expensive and we can use with off-label permission in recipients in our country because of financial reasons. Therefore, the duration of treatment is an essential issue. Studies also support long-term cinacalcet treatment of three and five years as effective and safe [26, 27]. On the other hand, some studies address the question of whether the effect of cinacalcet persists after withdrawal of the drug [15-18]. The optimal time point for a discontinuation is unknown. Cinacalcet treatment corrected hypercalcemia and decreased PTH levels in 10 kidney transplant recipients with hypercalcemia and severe PHPT, but re-elevated in two patients who discontinued cinacalcet [15]. In another study, serum Ca and PTH levels were increased in all participants after discontinuation of cinacalcet treatment for six months [16]. The authors concluded that continuation of long-term cinacalcet treatment is necessary to ensure normocalcemia. Kruse, *et al*. [17], evaluated serum Ca and PTH levels three months after cessation of a 1-year cinacalcet therapy. After withdrawal, serum Ca levels elevated immediately in all, but remained normal after three months. They argued if the normalization was an effect of cinacalcet or due to expected post-transplantation serum Ca decrease [17]. In our study, serum Ca levels increased significantly after cessation of cinacalcet in recipients within one month. Besides these compatible results, oppositely serum PTH levels unchanged, even slightly elevated, in our cohort under cinacalcet treatment, whereas serum Ca levels decreased. We thought that these results were associated with tertiary hyperparathyroidism because we showed adenoma-like hyperplastic glands in eight recipients; it was possible that the resistance was due to nodular hyperplasia in the remaining recipients. 

Resistance to cinacalcet is reported due to nodular hyperplasia of parathyroid gland in hemodialysis patients and recipients [28-30]. There are limited data about imaging of parathyroid gland and remains its mystery among recipients receiving cinacalcet. Okada, *et al. *[30], evaluated recipients under cinacalcet treatment with parathyroid USG, although USG were performed before transplantation. They reported that five recipients in whom USG revealed parathyroid gland enlargement were resistant to cinacalcet, and underwent PTx because of persistent hypercalcemia. Serum PTH levels did not only decrease but also elevated in their cohort; even their serum Ca levels remained high. In the present study, we evaluated all the recipients with USG and scintigraphy who were planned to receive cinacalcet. Parathyroid scintigraphy and USG revealed parathyroid adenoma in eight recipients. Seven underwent PTx with adenoma because of resistance to cinacalcet; the one who continued cinacalcet had contraindications for surgery. As in the previous study [30], recipients with adenoma are resistance to cinacalcet. Enlarged nodular hyperplastic parathyroid gland is as a predictor for cinacalcet resistance in recipients as well as in hemodialysis patients. There are monoclonal cell proliferation and low densities of calcium-sensing and vitamin D receptors in nodular hyperplasia [31-33]. As cinacalcet increases the sensitivity of Ca sensing receptors, low densities of these receptors explains cinacalcet resistance. Although in a rat model it was showed that cinacalcet attenuates parathyroid hyperplasia [34], significant enlargement and adenoma can be an obstacle. It is important to figure out the etiology of persistent hypercalcemia and decide whether it is due to secondary or tertiary hyperparathyroidism for choosing the appropriate treatment. In our study, it was considered that tertiary hyperparathyroidism was the leading cause for persistent hypercalcemia. Furthermore, histopathologic findings strengthen the opinion that the gland has gained autonomy. 

PTx should be considered to treat tertiary hyperparathyroidism. PTx ameliorates hypercalcemia permanently. Normal serum Ca levels were achieved with PTx in all our recipients in whom serum Ca levels re-elevated after cinacalcet withdrawal. However, worsening graft function, vocal cord palsy, serious hypocalcemia, and relapsing of hyperparathyroidism after PTx are reported concerns. Nonetheless, there were no significant adverse events after PTx in seven recipients in the present and other recipients in our previous study [7]. 

Our study had several limitations. Firstly, it was a small retrospective study. Secondly, lacking parathyroid gland volume and diameter assessments were important negative sides of this study. Undetailed USG evaluation might miss the possible gland hyperplasia in the resistant recipients without adenoma. With this respect, three recipients with normal parathyroid imaging had to undergo PTx because of unresponsive cinacalcet treatment. Nevertheless, the present study showed the association between parathyroid gland enlargement and the cinacalcet resistance in recipients and also the importance of imaging studies.

In fact, hyperparathyroidism should be effectively controlled to preclude gland enlargement in early stages of chronic kidney disease and before transplantation. In our recent study, we showed that graft function was significantly better in recipients who had PTx before transplantation compared with those either without PTx or who underwent post-transplantation PTx [7]. Candidates for transplantation should be evaluated for enlarged gland and, if necessary, PTx is better to be performed before transplantation to prevent negative effects on allograft. 

In conclusion, to think more accurately about the treatment option for persistent hypercalcemia in recipients, initially parathyroid gland should be evaluated with imaging studies. Recipients with enlarged parathyroid gland tend to have resistance to cinacalcet treatment, and PTx should be preferred under elective conditions. Cinacalcet can also be used untill PTx. Cinacalcet may be a more appropriate treatment for a sub-group of recipients with hypercalcemia without parathyroid adenoma or who have a contraindication for surgery. If serum PTH levels remain elevated with cinacalcet treatment in hypercalcemic recipients without adenoma, detailed imaging studies should be performed for enlarged glands. PTx should be considered in patients with adenoma or in resistant patients with elevated serum Ca and PTH levels despite cinacalcet treatment. Large-scale randomized controlled trials which are strengthened by imaging techniques are needed to evaluate the appropriate management approach for persistent hypercalcemia in kidney transplant recipients.

## CONFLICTS OF INTEREST:

**None declared.**

## FINANCIAL SUPPORT:

**None.**
